# Regulation of axon pathfinding by astroglia across genetic model organisms

**DOI:** 10.3389/fncel.2023.1241957

**Published:** 2023-10-24

**Authors:** Georgia Rapti

**Affiliations:** ^1^Developmental Biology Unit, European Molecular Biology Laboratory, Heidelberg, Germany; ^2^Epigenetics and Neurobiology Unit, European Molecular Biology Laboratory, Rome, Italy; ^3^Interdisciplinary Center of Neurosciences, Heidelberg University, Heidelberg, Germany

**Keywords:** axon guidance, glia-neuron interactions, development, embryo, model organisms

## Abstract

Glia and neurons are intimately associated throughout bilaterian nervous systems, and were early proposed to interact for patterning circuit assembly. The investigations of circuit formation progressed from early hypotheses of intermediate guideposts and a “glia blueprint”, to recent genetic and cell manipulations, and visualizations *in vivo*. An array of molecular factors are implicated in axon pathfinding but their number appears small relatively to circuit complexity. Comprehending this circuit complexity requires to identify unknown factors and dissect molecular topographies. Glia contribute to both aspects and certain studies provide molecular and functional insights into these contributions. Here, I survey glial roles in guiding axon navigation *in vivo*, emphasizing analogies, differences and open questions across major genetic models. I highlight studies pioneering the topic, and dissect recent findings that further advance our current molecular understanding. Circuits of the vertebrate forebrain, visual system and neural tube in zebrafish, mouse and chick, the *Drosophila* ventral cord and the *C. elegans* brain-like neuropil emerge as major contexts to study glial cell functions in axon navigation. I present astroglial cell types in these models, and their molecular and cellular interactions that drive axon guidance. I underline shared principles across models, conceptual or technical complications, and open questions that await investigation. Glia of the radial-astrocyte lineage, emerge as regulators of axon pathfinding, often employing common molecular factors across models. Yet this survey also highlights different involvements of glia in embryonic navigation or pioneer axon pathfinding, and unknowns in the molecular underpinnings of glial cell functions. Future cellular and molecular investigations should complete the comprehensive view of glial roles in circuit assembly.

## Introduction

Tracing back centuries, the complex architecture of nervous systems took center stage among biological tissues with remarkable heterogeneity and connectivity. Decades of investigations in diverse organisms exposed that the assembly of circuit architecture is driven by cellular and molecular events, often presenting a striking degree of conservation across vertebrate and invertebrate species. The mechanisms guiding neuronal processes include modes of contact-mediated or chemical attraction, contact-mediated or chemo-repulsion, that rely on membrane-bound or diffusible factors and provide permissive/ attractive or inhibitory/repulsive signals, respectively (Kolodkin and Tessier-Lavigne, 2011). Indeed, mathematical modeling proposes that the remarkable precision of pathfinding events *in vivo*, cannot arise solely from chemoattractive gradients (Goodhill, 2016). Fidelity may be ensured through synergies among attractive cues, avoidance of repellant boundaries, and other cellular mechanisms such as fasciculation with pioneer axons (Morales and Kania, 2017). Despite extensive studies in neurodevelopment, the number of known cues and mechanisms driving pathfinding appears small relative to the immense nervous system complexity.

During neural circuit formation, axons navigate over long distances and across complex and dynamic environments. They are thought to rely on intermediate choice points, to break their journey into smaller segments until reaching their final destination (Raper and Mason, 2010; Comer et al., 2019). This concept of “intermediate targets” emerged from seminal studies in the grasshopper limb bud (Ho and Goodman, 1982; Keshishian and Bentley, 1983) and is now generalized across various contexts and organisms (Avilés and Stoeckli, 2016; Frei and Stoeckli, 2017). Early descriptive studies in vertebrates highlighted the proximity of glial scaffolds and growth cones in the Newt spinal cord and proposed a “blueprint hypothesis.” This idea of a glial “blueprint” in the nervous system suggests that axon growth cones navigate in paths already populated by non-neuronal cells, such as glial cells or their neuroepithelial progenitors (Singer et al., 1979). Since the early observations of the nervous system architecture by Virchow, Ramón y Cajal, and Río-Hortega, neurons and glia were found intimately associated and their interactions were thought to be likely critical for formation and function of the nervous system (Ramón y Cajal, 1909; Shaham, 2005; García-Marín et al., 2007). Glial cells were long seen as faithful companions of neurons, albeit with a merely trophic function. However, it is now established that glial cells play key roles in neurogenesis, axon scaffolding, synapse formation, the formation of the blood–brain-barrier, and axon regeneration (Powell and Geller, 1999; Kettenmann and Verkhratsky, 2008; Kriegstein and Alvarez-buylla, 2011; Allen and Lyons, 2018). Glia could employ any of the above mechanisms of chemical or contact-mediated attraction/−repulsion, to drive stereotypical axon navigations during patterning, in addition to their roles in axon regeneration or growth *in vitro*.

While many glial cell types are generated after neurogenesis, some non-neuronal cells populate the nervous system before the navigation of early axon scaffolds. For example, neural progenitors (also referred as neural stem cells) are epithelial-like cells with bipolar morphology that transform into elongated radial glial cells with specialized endfeet during early neurodevelopment (Kriegstein and Alvarez-buylla, 2011). They reside in the ventricular and subventricular zones (VZ and SVZ) and are well-acknowledged for the generation of neurons and macroglia and the migration of neurons along their radial (basal) fibers (Gasser and Hatten, 1990; Noctor et al., 2002; Malatesta et al., 2008). Radial glia later trans-differentiate to generate astrocytes, through a division-independent morphogenetic transformation (Voigt, 1989). Radial glia and their astrocyte derivatives, while morphologically distinct, share some molecular characteristics, including expression of the Glial Fibrillary Acidic Protein (GFAP). Due to these molecular and lineage similarities, astrocytes and their progenitor radial glial cells are collectively referred to as *astroglia* (Campbell and Götz, 2002).

**Figure 1:**
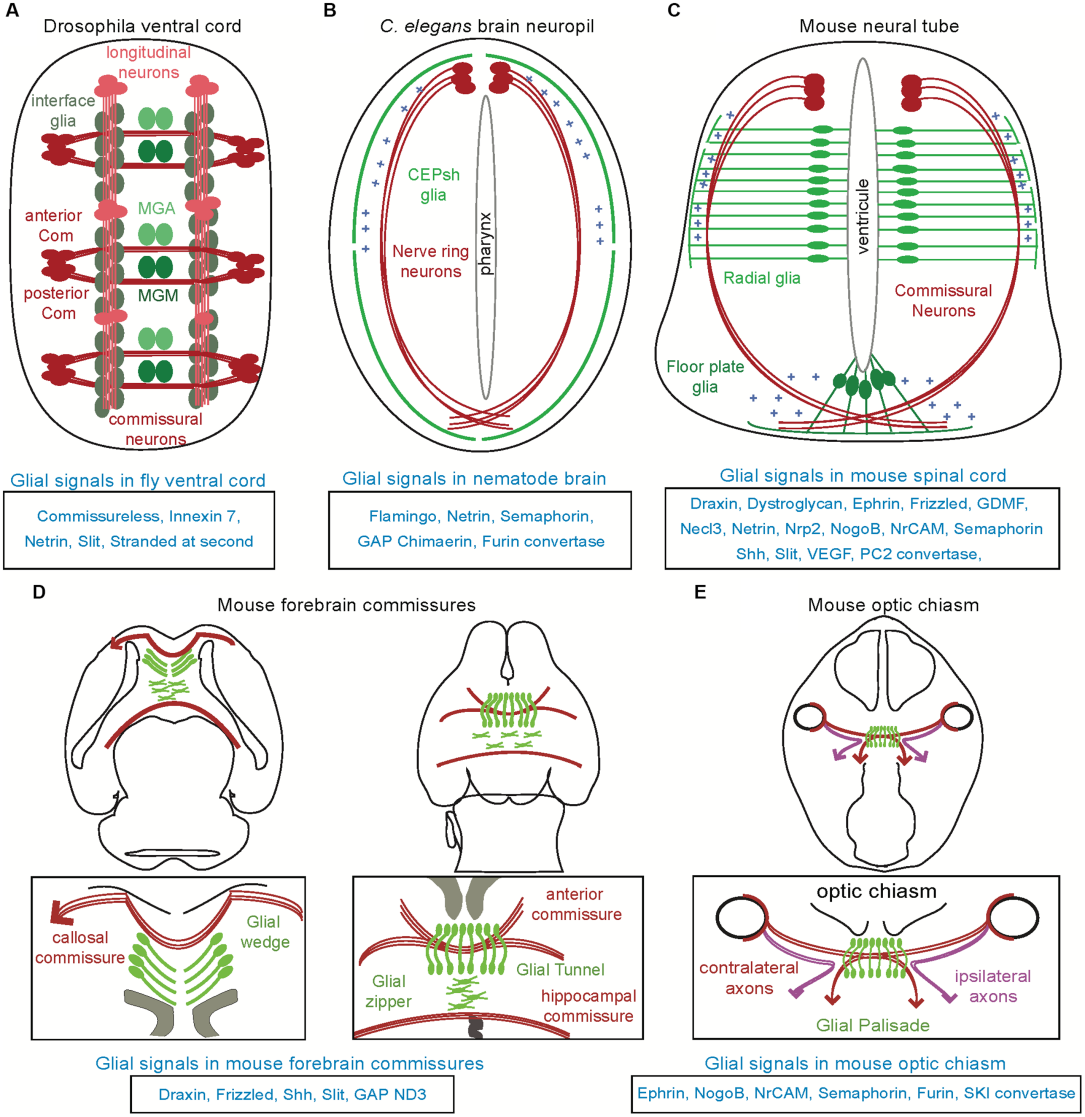
Distinct glial substrates appose to navigating axons in invertebrate and vertebrate models. Various types of glial cells (in different shades of green) appose to major axon commissures (in red), across different species. **(A)** In the ventral cord of *Drosophila*, the *midline anterior glia* (MGA) and *midline glia middle* (MGM) affect the commissural axon tracts while the interface glia affect the longitudinal axon tracts. **(B)** In the brain neuropil of *Caenorhabditis elegans* (“nerve ring”), the CEPsh glia drive pathfinding of brain-neuropil axons. **(C)** In the mammalian spinal cord, radial glia from the ventricular zone and the floor plate can regulate the pathfinding of commissural axons, and have similar effects in the chick (not shown here, see text). **(D)** In the mammalian forebrain, glial structures and their signals ensure proper navigation and formation of the callosal, anterior, hippocampal commissures. **(E)**. In the mammalian optic chiasm, glial cells are expressing various molecules acting to ensure proper axon pathfinding. Molecules underlying glia-mediated guidance are listed below each context.

Neural progenitors/radial glial cells and astrocytes (astroglia) appear to form regular physical boundaries throughout the nervous system which were hypothesized to affect early axon pathfinding, to prevent decussations and establish bilaterality of brain connections [see review by Chotard and Salecker (2004) and Stoeckli (2018)]. Neural/radial progenitors have been functionally implicated in axon guidance, in early sparse reports and numerous recent ones. They demarcate growing-axon paths in the peripheral nervous system (PNS) and principal axon commissures in the central nervous system (CNS). While this glia-mediated guidance activity was first reported for rather simple axon bundles in invertebrate organisms, it is recently described in many higher-order circuits and across all invertebrate and vertebrate models (Figure 1). In the last couple of decades, experimental observations across models suggest that axon growth and navigation guided specifically by astroglia may be an overarching mechanism initiating early assembly of circuit architecture throughout diverse centralized nervous systems.

**Figure 2:**
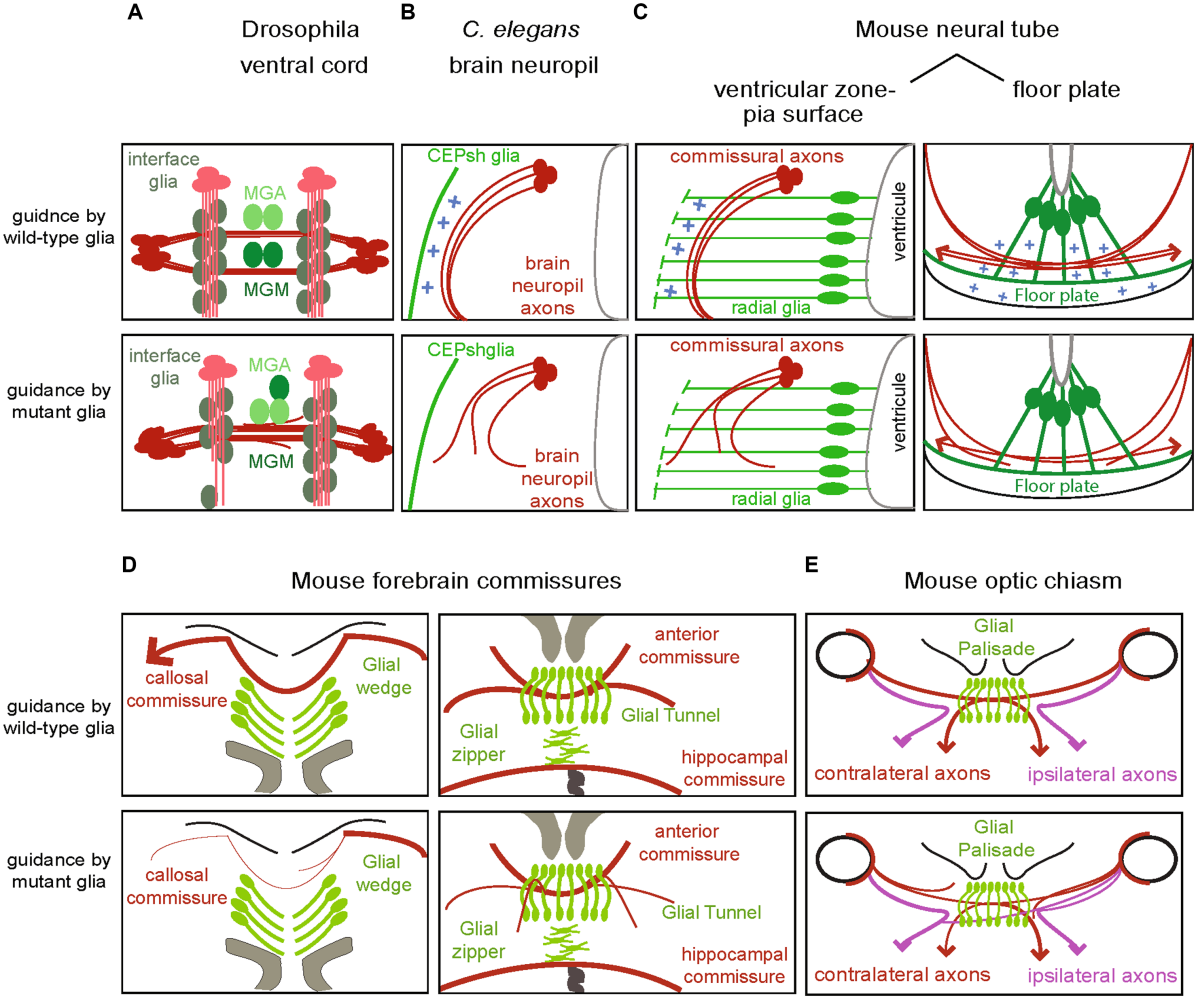
Disruption of fate or signals from glial substrates affect the associated navigating axons across models. **(A)** Longitudinal and commissural axons in the fly ventral cord suffer from abnormal navigation upon disruption of molecular signals of their associated glial cells, interface glia and midline, respectively (see text for details). **(B)** In the *Caenorhabditis elegans* brain neuropil, disruption of glial cues Netrin and Semaphorin or other cues results in ectopic navigation or abnormal termination of axons. **(C)** In the mammalian spinal cord, radial glia from the ventricular zone and floor plate regulate the pathfinding of commissural axons along the pia surface toward the midline and across the midline, respectively. Mutants of their homologs have similar effects in the chick (not shown). **(D)** Perturbing of any of the glial structures in the forebrain commissures, can result in dysgenesis of the corpus callosum or disrupt the formation of the anterior and hippocampus commissures. **(E)** Similarly, disrupting glial cell signal in the optic chiasm affect the trajectories of ipsi- and contra-lateral axons.

In this review, I present studies that highlight astroglial roles in axon pathfinding during development, including cases of contradictory findings in the field and possible reconciliation throughout the years. I survey how astroglial cells contribute to axon pathfinding during circuit assembly initiation, focusing on genetic model organisms that represent forerunners in the experimental efforts to understand mechanisms of circuit assembly *in vivo*. In the first section I present early studies that pioneered concepts of glial cell influence on axon navigation. In the following sections, I focus on different model organisms, starting with a brief summary of astroglial features. Then I outline glia–neuron appositions (Figure 1) and glial-mediated mechanisms guiding axons for the assembly of circuit architecture (Figure 2; Table 1). I focus on direct regulation of axon pathfinding and not the regulation of axon architecture through glial wrapping/ myelination or engulfment. I emphasize pathfinding mechanisms of early circuit assembly and do not outline cases of regenerating axons that navigate in pre-existing circuits. I focus on informative studies that establish glial roles in axon pathfinding *in vivo* but do not present *in vitro* studies comprehensively. When possible, I try to maintain a largely chronological order of studies within one circuit and organism, to highlight the advancement of concepts throughout time, in different models. I finish by briefly discussing possible overarching themes of the astroglia-mediated guidance and its involvement in diseases of neurodevelopment as well as highlighting open questions awaiting future investigation.

**Table 1:** Summary of key proteins underlying glial cell-regulation of axon growth/pathfinding.

Glial proteins	Glial cells of model organisms					Neuropil
	*Mouse*	*Chick*	*Zebrafish*	*Drosophila*	*Caenorhabditis elegans*	CNS/PNS
**Guidance cues/morphogens**						
Commissureless				VC midline glia		Ventral cord
Draxin	Floor plate, Forebrain midline glia,	Floor plate				CNS vertebrate forebrain and spinal cord commissure
Dystroglycan	Floor plate					CNS spinal cord
Ephrins	Floor plate, optic chiasm glia					CNS spinal cord, optic chiasm
Flamingo				Brain surface/cortex glial	Brain astroglia	Fly photoreceptor neurons, nematode brain
Frizzled		Floor plate	forebrain midline glia			CNS forebrain, spinal cord
GDNF	Floor plate					CNS spinal cord
Innexin 7				VC interface glia		Ventral cord
Insulin-like peptide dilp6				Brain surface glia, cortex glia		Brain, insulin-producing cells (IPCs)
Necl3/SynCAM2		Floor plate				CNS spinal cord
Netrin	Floor plate glia, ventricular zone RGs, forebrain midline glia			VC midline glia	Brain astroglia	CNS vertebrate forebrain and spinal cord, fly ventral cord nematode brain
Neuroglian				Brain TIFR glia, PNS support cells		Brain IPCs, PNS DA neurons, ORNs
Neuropilin Nrp2	Floor plate					CNS spinal cord
Nogo-B	Floor plate, optic chiasm glia					
NrCAM	Floor plate, optic chiasm glia	Floor plate				CNS spinal cord
Semaphorins	Floor plate, optic chiasm, olfactory ensheathing cells	Floor plate		Brain glia	Brain CEPsh astroglia	CNS vertebrate spinal cord, nematode brain
Shh	Floor plate		Forebrain midline			CNS forebrain
Slit(s)	Floor plate, brain glial wedge and indusium griseum	Floor plate	Forebrain midline	VC midline glia		CNS vertebrate brain and spinal cord, fly ventral cord
Stranded at second				VC interface glia		Ventral cord
VEGF	Floor plate					CNS spinal cord
Wnt inhibitor Frzb	Olfactory ensheathing glia					olfactory sensory neurons

**Transcription factors in glia mediating guidance**							
	*Mouse*	*Chick*	*Zebrafish*	*Drosophila*	*Caenorhabditis elegans*	CNS/PNS	
Lhx2	Glial wedge, indusium griseum glia		Spinal cord glia			CNS: mouse brain, zebrafish ventral cord	
NKX2	Ventral telencephalon glial cells		Ventral spinal cord glia		Brain CEPsh astroglia	CNS: mouse brain, zebrafish ventral cord, nematode brain	
**Other proteins**							
GAP of ND3	Medial ganglionic eminence RGs					Corticospinal axons	
GAP Chimaerin					Brain astroglia	Nematode brain	
Mmp2 protein convertase				Exit glia		PNS motor axons	
Protein convertases (Furin/SKI/PC2)	Optic chiasm radial glia	Floor plate			Brain astroglia	CNS mouse spinal cord, optic chiasm axons, nematode brain	

The column I provides information on guidance cue protein name-type. The column II-III-IV-V-VI note the model organisms populated by astroglial cells mediating axon navigation, the column VII provides information on the CNS/PNS neuropil the glial cells are acting for guidance (CNS, Central Nervous System; PNS, Peripheral Nervous System; RG, radial glia, VC, ventral cord, TIFR, Transient-Interhemispheric-Fibrous-Ring, DA, dendritic arborization, ORNs, olfactory receptor neuron; GAP, GTPase-activating protein).

## Pioneering the concept of glia-mediated axon guidance

Some of the first functional evidence that glial cells can act as functional guideposts for growth cone guidance was provided by experiments in insects. In the grasshopper limb buds, embryonic U axons contact the *glial cell segment boundary cell* (SBC) and exit the ventral nerve cord to pioneer the *intersegmental nerve* (IS), followed by the growth cone of the aCC motor neuron. Upon laser ablation of the SBC glial cell, the U and aCC growth cones continue past its normal exit point, suggesting a glial role as guidepost cells for the IS nerve pioneer and follower neurons (Bastiani and Goodman, 1986). More recently, the presence of the SBC glial cells as a turning point to the IS nerve was also shown in *Drosophila*, together with other classes of glial cells present in major axon pathways (Rigby et al., 2020). Early molecular studies in the *Drosophila* also identified that the extracellular protein Slit is expressed by fly midline glia, and is required for the development of midline glia and the guidance of commissural axons in the fly ventral cord (Rothberg et al., 1990). In mammals, ultrastructural studies in the sensorimotor cortex of hamsters highlighted that axons of callosal afferents are tightly associated with radial glia processes and their growth cones did not extend beyond associated radial glia fibers (Norris and Kalil, 1991). Such early descriptions of glial cells’ associations with axon paths were suggestive of glial roles in axon guidance in different contexts. In later years and until today, studies combining descriptions of cellular and molecular interactions implicate glial cells in the regulation of axon development.

## Axon guidance by glia: insights from *Drosophila*

Insect glial cell types, categorized according to their positions, include *cell-body-associated, neuropil-associated, and surface-associated glia* in the CNS, and *nerve-associated glia* in the PNS. Glial cells in *Drosophila* comprise only 10% of all CNS cells and yet they display surprising morphological and functional diversity. Two morphologically defined glial cell types named *astrocyte-like* and *cortex glia,* display astrocytic functions. Cortex glia associate with neuronal cell bodies and synapse-free proximal neurites and provide trophic support to neurons, while the fly astrocyte-like (or astrocyte) glial cells extend processes in the neuropil to interact with synapses (Chotard and Salecker, 2004; Nagai et al., 2021). The astrocytes are the most well-studied insect glia and regulate axon guidance among other neurodevelopment processes. Other glial cells are positioned as midline cells across various axon paths in the fly nervous system. A common organizational principle in Bilateria, including arthropods and chordates, is that two halves of the CNS connect by axon commissures of interneurons projecting across the midline, where the two lateral neurogenic regions are separated by midline cells. These midline cells play a key role in guiding commissural axons, both in fruit flies and vertebrates. Such roles of fly glial cells were first studied in the fly ventral cord and later in the brain and PNS.

### Glia-mediated guidance in the *Drosophila* brain neuropil

The ventral nerve cord of the fly is characterized by two longitudinal axon bundles (also known as *longitudinal connectives*) that run along the length of the embryo and two commissural fascicles crossing the midline that join in each segment, in a ladder-like fashion. Since the late 1990’s, different parallel studies implicated glial cells in the formation of both the commissural axons and longitudinal tracks (Figure 1A).

Seminal studies in *Drosophila* ventral cord hypothesized that *midline glia* were important for commissural axon pathfinding, and demonstrated that abnormalities in the differentiation and migration of these glia correlate with axon misrouting and commissural mis-fasciculation (Klämbt et al., 1991). The first growth cones pioneer the posterior commissure of the ventral cord, by extending toward the posterior edge of the midline glia MGA (*midline glia anterior*; Figure 1A). They then continue to pioneer the anterior commissure, that closely associates with the posterior commissure at the midline. No glial migrations are observed at that time. While these commissures initially fasciculate posterior to the MGA glia, the separation of these commissures correlates with migration of the glia MGM (*midline glia middle*). Glia migrations complete by the time the longitudinal connectives are formed. In certain mutations that affect the differentiation or the migration of midline cells, commissures may begin to form abnormally but later atrophy and disappear or do not separate and remained fused. Thus, the fly midline glia appear to be dispensable for the initial formation of the posterior commissure but play a role in the development of the anterior commissure and the later separation of the commissures (Figure 2A).

Early studies also proposed glial roles for axon pathfinding in the longitudinal axon tracts of the fly ventral cord. Longitudinal axons tracts form by interneuron axons that fasciculate with pioneer axons to grow along the longitudinal pathways connecting to the brain (Figure 1A). An array of interface glia foreshadows the scaffold of longitudinal axon tracts, and is suggested to help in establishing these longitudinal tracts (Hidalgo et al., 1995). Contrary to commissural neurons, the axons pioneering the longitudinal pathways (pCC, MP1, dMP2, and vMP2) lack any midline crossing but extend and turn toward longitudinal glial cells, that form a scaffold prior to axon growth. Cell ablations of longitudinal glia appear to affect longitudinal axon fasciculation, while their requirement for growth cone guidance is not absolute (Hidalgo et al., 1995). Moreover, killing the glia after pioneer neuron formation disrupts the pathfinding of follower axons, suggesting that these glial cells cooperate with pioneer axons to direct the follower growth cones. Yet, the role of glial cells in *Drosophila* pathfinding is somehow controversial. *Glial cells missing (gcm)* mutations, that alter glial cell fate, appear to alter the formation of some longitudinal tracts while others can still form, thus questioning the glial cell requirement for axon pathfinding. However, the *gcm* mutation is proposed to transform only some glia and maintain some glial features, including their division and migration patterns, thus possibly not corresponding to the lack of all midline glia (Hosoya et al., 1995; Jones et al., 1995; Hidalgo and Booth, 2000).

These early descriptive studies dissected glia–neuron associations in remarkable single-cell resolution, including in genetic mutants. However, some midline cells associating with commissural abnormalities are neurons (RPI), possibly obscuring the glial-specific effect. Moreover, glial-specific interactions with axons and the underlying molecular mechanisms remained unknown at first. Within a few years of these descriptive studies, Netrin and Slit-Robo signaling were suggested to act in midline glia for commissural guidance (Table 1). Netrins, required for commissural axon guidance in the fly midline, are expressed in midline glia MGA and MGM and a few midline neurons (including neuroblasts and VUM neurons; Harris et al., 1996; Mitchell et al., 1996). *Drosophila* Netrins act through short-range interactions with the Frazzled receptors (Bashaw and Goodman, 1999; Brankatschk and Dickson, 2006) and cooperate with neuronal Flamingo (Organisti et al., 2015). More recently, Netrin of the ventral neuroectoderm was implicated in the migration of glia or their precursors (Von Hilchen et al., 2010). Thus, whether fly Netrin functions as a glial-derived guidance cue or it guides migration of glial cells which then affects axon pathfinding is not clear. Recently, the expression of Netrins in midline glia, and its function in glial cell migration and axon patterning was also shown in the ventral cord of spider species (Linne and Stollewerk, 2011).

In addition to Netrin, midline glia also express the extracellular matrix protein Slit to repel commissural axons after midline crossing (Table 1). Slit can bind to Robo, an immunoglobulin superfamily receptor highly expressed on axon growth cones of commissural axons (Tuyen et al., 1999). In Robo and Slit mutants, axons aberrantly (re)cross the midline suggesting that Slit is a glial cue that regulates axon repulsion via binding to neuronal Robo receptor, which acts cell autonomously. However, Slit is also implicated in the development and migration of glial cells prior to pathfinding of midline commissural axons, raising the question about its primary contribution in glial migration or axon guidance (Rothberg et al., 1990; Von Hilchen et al., 2010). The transmembrane protein Commissureless can also act in midline glia and commissural axons prior to midline crossing, to downregulate surface levels of axonal Robo1 and block premature responsiveness of axons to the midline repellent Slit (Tear et al., 1996; Georgiou and Tear, 2002). Moreover, another pathway has evolved to overcome Slit-Robo1 repulsion in pre-crossing commissural axons: fly midline glial cells also express Robo2 receptor which interacts in trans with Robo1 on pre-crossing axons to prevent canonical Slit–Robo1 repulsion (Evans et al., 2015). Robo2 is expressed in both midline glia and neurons and thus its glial roles remain to be better distinguished in this context.

More recent studies also contribute to the molecular understanding of how glial cell signaling drives differential axon pathfinding of commissural and longitudinal paths in the fly ventral cord. Innexin 7 acting in midline glia can affect intrasegmental fusion of the ventral cord and the formation of the longitudinal, posterior, and anterior commissures (Ostrowski et al., 2008). Whether loss of Innexin 7 affected specifically the glial communication with axons or the morphology of midline glia was not investigated but it appears key since recent studies implicate connexins (innexins’ orthologs) in vertebrate astroglial morphology (Ghézali et al., 2018). Glial-axon communication underlying axon pathfinding in the midline also involves s-Ptp10D protein interactions (Lee et al., 2013). The cell-surface protein Stranded at second (Sas), expressed in midline glia, binds to the neuronal type III Receptor Tyrosine Phosphatase Ptp10D, to regulate glia morphology and subsequent axon pathfinding in the midline. The loss of Sas or Ptp10D disrupts midline glial cell morphology, and redundantly with Ptp69D affects the decisions of axon growth cones to choose longitudinal vs. commissural pathways.

Thus, glial cells of the fly ventral cord appear to drive attraction and repulsion of commissural and longitudinal axons toward and away from the midline, through the conserved signaling pathways of Netrin and Slit-Robo, respectively. Other conserved cell-surface proteins including innexins and Receptor Tyrosine Phosphatases provide additional regulation of glia-mediated axon pathfinding.

### Glia-mediated guidance in *Drosophila* brain neuropils

Whether glia help axon pathfinding in higher-order fly brain neuropils remained for long unclear (Pfrieger and Barres, 1995) and was explored more in the past dozen of years. Formation of the embryonic brain neuropil (*protocerebrum*), begins when neuroblasts delaminate from the neuroectoderm and generate 10–20 primary neurons each. Axons emitted from same-lineage neurons fasciculate and form the primary axon tracts (PATs), which arborize to generate a stereotypical set of neuropile compartments in the late embryo. Cortex and neuropile glia ensheath these axons later, but form after the embryonic PATs and may not contribute to pioneer axon guidance in the embryonic brain. Instead, the formation of the protocerebrum appears to depend on neuron–neuron interactions via the Slit/Robo signaling. While Slit from midline glia drives axon guidance in the embryonic fly ventral cord, the protocerebrum is thought to not contain midline sources expressing Slit. The neurons of the mushroom body (MB) are proposed to be the major source of Slit in the fly protocerebrum. The MB neurons express Slit but not Robo receptors, suggesting that they may drive neuropil formation through neuronal communication with surrounding neuropil areas (Oliva et al., 2016). Conversely to neuron-mediated axon guidance in the embryonic brain, brain glial cells appear to guide the larval secondary axon tracts (SATs), cohesive axon bundles of same-lineage neurons generated by larval mitosis of neuroblasts (Younossi-Hartenstein et al., 2003, 2006; Pereanu et al., 2005). Glial cells, born concurrently with pioneer neurons, form segmentally iterated landmarks of elongated tracks. SATs interact in a characteristic way with glial cells while traversing the brain neuropile and require cortex and neuropile glia for proper growth and targeted fasciculation, as suggested by cell ablations (Spindler et al., 2009). The glial molecules driving axon guidance of SATs in the fly larval brain remain largely unknown.

More recently, glial cells were also implicated in the pathfinding of R8 photoreceptor neurons in the medulla of the larval visual system as well as the insulin-producing cells (IPCs), a group of 14 neurons in the *Drosophila* larval brain. R8 axons pioneer the formation of the medulla columns in the optic ganglia, which preserve the relationship between the visual world and its brain representation (Zipursky and Sanes, 2010). R8 axons project to a single column without bundling with each other and then extend to the M3 layer, through interactions between transmembrane proteins Golden goal (Gogo) and atypical cadherin Flamingo. While Gogo and Flamingo cooperate early to guide the R8 inside the column, Gogo is later phosphorylated to counteract Flamingo and suppress filopodia extension and finally ceases to express, allowing Flamingo to direct R8 axons to the M3 layer (Table 1). The switch of Gogo function from cooperative to antagonistic toward Flamingo is regulated through its phosphorylation by the insulin receptor. Surface and cortex glial cells provide insulin-like peptide DILP6 to activate the insulin receptor for Gogo phosphorylation. Moreover, glial protrusions present Flamingo that interacts with Flamingo in the R8 neurons to regulate pathfinding (Takechi et al., 2021).

The *insulin-producing cells* (IPCs) navigate following glial signaling by the adhesion molecule Neuroglian (Nrg), a homolog of the vertebrate cell adhesion molecule L1, the guidance cue Semaphorin-1a and receptor PlexinA (Clements et al., 2021). These 3 factors are expressed and required in both glial cells and IPCs to control for IPCs axon growth, branching, and fasciculation, albeit with a stronger contribution of Nrg and Sema-1a in glia and PlexA in the IPCs. Nrg, acting from glia as a suppressor of neurite sprouting of IPCs, was also previously implicated in guiding olfactory receptor neurons (ORNs) to generate the odortopic map in brain glomeruli (Chen and Hing, 2008). ORNs, growing stereotypically from peripheral organs, bifurcate to innervate both ipsilateral and contralateral antenna lobes by converging their axons onto the dendrites of projection neurons. The Transient-Interhemispheric-Fibrous-Ring (TIFR) glia closely associate with ORN axons during their development and express Nrg, which regulates glial morphology and midline crossing of ORNs. Yet, the glial cell expression of Nrg is not sufficient in this context, suggesting Nrg may be required from additional cells to guide the antennal commissure.

### Glia-mediated guidance in the *Drosophila* PNS

Glial cells are also implicated in neuronal pathfinding in the embryonic PNS. In the visual system, glial cells are first to colonize the lamina before neuron differentiation occurs, they establish the distinct epithelial and marginal layers and then direct photoreceptor axon targeting. They are suggested to provide unknown local, adhesive stop-signals to prevent axons entering the medulla (Poeck et al., 2001). Upon innervation of the fly epithelia, the *dendritic arborization neurons (DA)* neurons depend on glial cells for their proper branching, via the function of Neuroglian in glia and neurons (Miller et al., 2011). Moreover, motor neurons interact with *exit glia* expressing membrane-associated Mmp2 to regulate axon targeting and fasciculation by controlling levels of the ECM protein Frac (Yamamoto et al., 2006). Upon Mmp2 regulation, Frac expressed by mesoderm adjacent to axons and Mmp2-positive glia, controls a LIM kinase 1-dependent BMP signaling for proper embryonic motor axon pathfinding.

In summary, *Drosophila* studies have contributed pioneering ideas and descriptions about glial-mediated axon pathfinding. The fly glial cells have been implicated in axon pathfinding in several contexts of the *Drosophila* CNS and PNS. In the CNS fly ventral cord, commissural axons are regulated by MGA, MGM glia and longitudinal axons by longitudinal glia, while in the larval (but not the embryonic) fly brain the secondary axon tracts are regulated by cortex and neuropile glia. In the fly PNS, photoreceptor axons are guided by glia, TIFR glia guide axons of olfactory neurons and exit glia affect motor neuron axons. Importantly, conserved pathways of Netrin, Slits, Semaphorins, Flamingo as well as innexins and insulin signaling have been involved. In some cases, the underlying molecular pathways and the glial cell contributions in single-cell resolution remain to be elucidated.

## Glia guidance of brain assembly: insights from *Caenorhabditis elegans*

The nematode *C. elegans* provides an insightful setting to investigate astroglia. Its nervous system and connectome are mapped and are composed of 302/ 391 neurons and 56/ 92 glia, in hermaphrodite/ male animals, respectively (White et al., 1986; Cook et al., 2019). Its glial cells remained understudied until the last two decades when they started being molecularly and functionally investigated and compared to vertebrate macroglia (Shaham, 2005). Today *C. elegans* offers an array of approaches for *in vivo* imaging and molecular genetics, allowing manipulation and visualization of glial cells and neurons in single-cell resolution, as reviewed by Rapti (2020) and Singhvi et al. (2023). Among the *C. elegans* 56 glial cells there are 4 glial cells similar to vertebrate astroglia, the only ones associated with brain axons from the 52 neuroectoderm-derived glia. These CEPsh (*CEPhalic sheath*) glial cells grow radial-like membrane processes in the embryo, to later undergo a division-independent transformation and give rise to ramified astroglia (White et al., 1986; Rapti et al., 2017). This process is reminiscent of the transformation of mammalian radial glia to astrocytes. The *C. elegans* Olig2 transcription factor is expressed in these glial cells, while its homolog is expressed in certain mouse astrocytes, and homologs of the NKX transcription factor family specify the fate of CEPsh and forebrain astrocytes neighboring the anterior commissure (Yoshimura et al., 2008; Minocha et al., 2015). Moreover, postembryonic CEPsh glial cells are more similar to mouse astrocytes than to other brain cell types, based on quantitative analysis of transcriptomics (Katz et al., 2019). Remarkably, postembryonic CEPsh glial cells ensheath with their membrane endfeet the whole brain neuropil as well as individual synapses (White et al., 1986), to regulate synaptic morphology and neurotransmission (Colón-Ramos et al., 2007; Katz et al., 2018, 2019).

These glial cells have important roles in the pathfinding of axons in the brain-like neuropil (Rapti et al., 2017). They grow early embryonic, radial-like membrane processes that coalesce with pioneer axons of defined identity and guide them by the use of the secreted guidance cue Netrin (Figure 1B). In addition to Netrin, these glial cells employ the secreted cue Semaphorin to guide follower axons of different neuron modalities, including interneurons and sensory neurons (Rapti et al., 2017; Figure 2B). They also cooperate molecularly with pioneer neurons to guide follower axons, through the use of the conserved factors Flamingo, Furin, and Chimaerin (Rapti et al., 2017). Furin and Chimaerin appear to be acting upstream of glial guidance cues and affect their trafficking. On the other hand, CNS axons of the ventral cord and most other PNS axons do not appear associated with glial cells and their navigation follows cues provided by epithelial cells, as reviewed in Singhvi et al. (2023). Other glial cells in the *C. elegans* male tail may also associate with axons but their actions in morphogenesis remain understudied. Interestingly, the *C. elegans* brain neuropil resembles the developing mouse neural tube in terms of glial-mediated axon navigation; in both contexts, a circumferential ring of axons grows dorsoventrally toward the midline and glial cell endfeet demarcating the outer part of this axonal ring guide its path (Figure 1B). Moreover, the aforementioned cues driving glial-mediated guidance in *C. elegans* are conserved and their homologs are employed in many invertebrate and vertebrate contexts of axon navigation, albeit not always recognized for glial cell functions. Mouse Netrin and Semaphorin are provided by radial glial cells of the ventricular zone and the floor plate midline structure respectively, to guide commissural axons in the developing spinal cord while mouse Furin convertase appears expressed in the radial glial of the optic chiasm and acts for processing guidance cues (Kuwajima et al., 2012; Dominici et al., 2017; Varadarajan et al., 2017; see below). Importantly, glial mechanisms of axon pathfinding are underscored by significant cellular and molecular redundancies in *C. elegans* (Rapti et al., 2017) and in mammals (Wu et al., 2019). *Caenorhabditis elegans* provides an excellent genetic model to uncover hidden factors of such redundancies through modifier screens (Rapti et al., 2017). Overall, the *C. elegans* CNS provides a simplified but important setting to study glia–neuron interactions for circuit assembly, with brain glia similar to mouse radial glia/astroglia and glial mechanisms with putative conservation in vertebrates.

## Axon guidance by astroglia: insights from zebrafish

Within vertebrate systems, zebrafish is a key model that provides evidence on the cellular interactions and molecular mechanisms of glial cells directing axon pathfinding. It combines transparency for *in vivo* imaging and the possibility of genetic investigations. Nowadays, two astroglial cell types are recognized in zebrafish, *radial astroglia/ astrocytes* and *astrocytes*, after a long-standing belief that astrocytes were not present. The radial astroglia/ astrocytes are GFAP-positive glia with radial morphology and capacity of neuroglia progenitors throughout life and are described to sometimes display astrocyte markers and process branching associated with neurons (Lyons and Talbot, 2015). Otherwise, recent studies described zebrafish spinal cord astrocytes analogous to mammalian astrocytes, with shared molecular markers, tilling, neuron association and circuit function (Nagai et al., 2021). Functional studies of zebrafish glial cells often focus on the regulation of circuit function or axon navigation during post-injury regeneration. Certain studies summarized here provide insights about possible glial roles in the early neurodevelopment and patterning of circuit architecture.

### Glia-mediated guidance in the zebrafish CNS brain commissures

Early studies in zebrafish described characteristic positions of astroglia or their fibers, forming structures between each center and border region of the hindbrain rhombomeres and aligning to the forebrain post-optic commissure (POC), the anterior commissure (AC) and the optic nerve (Trevarrow et al., 1990). Similarly with the mouse cortex, zebrafish presents astroglia with cell bodies positioned ventrally and extend radial processes dorsally, terminating with endfeet in the pial surface. A small population of glia, expressing a GFAP homolog, spans the forebrain midline in characteristic positions and prior to the axon crossing of anterior and post-optic commissure. In mutants of the transcription factor Lhx2 these glial cells are misplaced and commissural axons associate with them and fail to cross the midline (Seth et al., 2006). Yet, it remains unclear which of these glial or axonal defects precede or follow. The correct positioning of glial cells and commissure axons also depends on Shh, Frizzled, and Slits signals. Here, in contrast to a direct axon guidance role, Shh is necessary to establish a proper expression pattern of the cues Slit2 and Slit3, that together with Frizzled-3a control patterning of midline glia (Barresi et al., 2005; Hofmeister et al., 2012). These midline glia express Slit1, which channels the forebrain axonal commissures by a permissive or positive mechanism (Barresi et al., 2005). Moreover, Olig2+ glial cells of non-radial glial lineage in the telencephalon and of radial glial lineage in the diencephalon have been suggested to interact with developing commissures (Schnabl et al., 2021).

An array of additional zebrafish genes were recently proposed to affect glial development in the forebrain/hindbrain/spinal cord and their associated axons (Barresi et al., 2010). Disruption of protein phosphatase 1, MAK16 homolog and a POU domain-class 5-transcription factor, an U3 small nucleolar ribonucleoprotein, an aryl hydrocarbon receptor nuclear translocator or the Wnt-family member 5b, the mind bomb E3 ubiquitin ligase, the TWIST neighbor, or the neuronal cadherin 2 correlates with abnormal placement, disorganization or abnormal morphology of certain forebrain and/or hindbrain and/or spinal cord glia. These defects in glial cell number, positioning, or structure also correlate with defects of axon wandering or de-fasciculation in the post-optic and anterior commissures. While it remains unknown if underlying genes primarily act in glia/axonal development or glia–neuron communication, the tight association of glial cell and axon defects suggests that distinct zebrafish glial populations may affect pathfinding decisions of various commissures.

### Glia-mediated guidance in the zebrafish PNS

In the zebrafish PNS, glial cells are suggested to assist the formation of axon patterns through barrier roles and fasciculation, but direct axon guidance roles by glia were not elucidated. In the CNS-PNS borders in Zebrafish, nkx2.2a-positive glial cells, born in the ventral spinal cord and migrating in the periphery, form the perineurium ensheathing motor nerves and Schwann cells. These *perineurial glia* have barrier and guidance functions at exit points of the spinal cord, ensuring motor neurons and axons do not migrate inappropriately outside of the spinal cord (Kucenas et al., 2008). In the zebrafish peripheral lateral line, axons and neural crest-derived peripheral glia migrate in a completely synchronized manner. Lateral line glial precursors are important to maintain fasciculation of the mature lateral line nerve but they appear dispensable for initial axon pathfinding. Here again, axons appear to instruct glia migration (Gilmour et al., 2002).

While little is known about the astroglial signals regulating specifically axon assembly in zebrafish, certain studies highlight axon decisions that can proceed independently of glial cell signals or contacts. At the PNS/CNS interface, pioneer axon growth cones of PNS sensory neurons appear to not contact glial cells when reaching the dorsal root entry zone (DREZ), and crossing to enter the CNS (Nichols and Smith, 2019). Yet it is described that these axons initially extend together with glial cells and the molecular mechanisms of pioneer axon initiation and extension through the DREZ remain to be characterized. In the zebrafish retina, *Müller glial cells* were initially suggested to be required for retina layer formation (Willbold and Layer, 1998; Bernardos et al., 2005). However, recent studies suggest that in the absence of proper Müller glial cell differentiation the retinal neuropil can still form with a relatively normal sublaminar organization (Randlett et al., 2013). Opposing results in different studies may relate to the specific genetic tools used to inactivate glial cells or molecules. While identifying glial roles requires manipulations that maintain other non-glial cell types intact, ruling out glial roles by causing complete glial disruptions requires to ensure that no cells with intermediate glial-like fate or partial signaling remain after the manipulations.

## Axon guidance by astroglia: insights from the mouse

Mouse glial cells populate almost half of the mouse nervous system and present a variety of cell types with distinct morphological, molecular, and functional characteristics (Powell and Geller, 1999; Kettenmann and Verkhratsky, 2008; Kriegstein and Alvarez-buylla, 2011; Allen and Lyons, 2018). Radial glial cells, astrocytes, and oligodendrocytes are well-known types of ectoderm-derived glial cells. Radial glial cells are the progenitors of the two latter, they generate oligodendrocyte precursors by division and then astrocytes by a division-independent trans-differentiation (Voigt, 1989; Noctor et al., 2002; Malatesta et al., 2008). Radial glial cells and astrocytes (collectively termed *astroglia*) are shown to interact with axons growing and navigating during development in different parts of the mouse CNS and PNS. Astroglial cell appositions to axon commissures were described early in the forebrain commissures and the optic nerve as well as the developing spinal cord.

### Guidance by glia in the mouse forebrain commissures

The major axon commissures in the mouse forebrain are the anterior and posterior commissure, corpus callosum, hippocampal commissure, and habenular commissure. These commissures have long been shown to spatially associate with boundaries between neuromeres presenting characteristic midline glial populations (Figures 1C–E). Midline glial populations in the embryonic dorsomedial cortex, known as the *indusium griseum, glial wedge, and midline zipper*, are populated by radial glia retracting their apical endfeet and translocate to the overlaying pia (Figure 1). These glial structures are closely positioned to major axon paths since early development, leading to propose that they may provide a preformed pathway for axon guidance (Levitt and Rakic, 1980; Van Hartesveldt et al., 1986; Silver et al., 1993; Marcus and Easter, 1995; Cummings et al., 1997; Fitch and Silver, 1997; Pires-Neto et al., 1998).

The corpus callosum commissure is a major connection of roughly 190 million axons that relays neural information between the two vertebrate brain hemispheres and supports cognitive functions (Tomasch, 1954). During the formation of the corpus callosum, cortical axons from one brain hemisphere cross the midline to reach their targets in the opposite cortical hemisphere. A cellular scaffold that was early implicated in axon guidance of the corpus callosum commissure is the subcallosal sling known as “*glial sling.”* The *sling* was thought to be composed of glial cells, classified as glioblasts rather than mature astrocytes. Its disruption was shown to prevent growth of callosal axons across the midline (Silver et al., 1982). However, the sling was recently shown to be largely composed of neurons (Shu et al., 2003). Thus, the sling-mediated mechanisms of callosal axon pathfinding may not be mediated by glial cells. In addition, developing callosal axons are shown to grow between the structures of *glial wedge* and *indusium griseum,* avoiding both of them (Figures 1D,E), and were also thought to recognize the structure *midline zipper* as guidepost cells (Silver et al., 1993). These glial cell populations have a different origin from the sling cells, derived from the subventricular zone (Shu and Richards, 2001). Disrupting the formation of these midline structures affects the formation of corpus callosum and hippocampal commissures *in vivo* (Figure 2D). Glia-specific disruption of the transcription factor Lhx2, expressed in the *glial wedge*, results in abnormal cell-cycle exit, defective formation of the *glial wedge* and subsequent agenesis of the corpus callosum (Chinn et al., 2015). Lhx2 disruption appears to also affect the structure of *indusium griseum*. Proper glial cell translocation and formation of the *indusium griseum*, *glial wedge* and *midline zipper* also depends on fibroblast growth factor receptor 1 (Fgfr1). Glial-specific loss of Fgfr1 causes disrupted glial structures, and subsequently affected the corpus callosum and hippocampal commissure (Ohkubo et al., 2006). Importantly, neuron-specific deficiency in Lhx2 or Fgfr1 appear to have normal corpus callosum and midline structures.

The midline structures apposing to the callosal commissure are shown to express secreted guidance cues such as the chemorepellent Slit2, which can guide the Robo1/2-expressing callosal axons (Shu and Richards, 2001). Formation of the *glial wedge* and *indusium griseum* as well as its expression of Slit2, encountered by callosal axons, is also regulated by the tumor suppressor Nf2 through suppression of the transcriptional coactivator Yap (Ohkubo et al., 2006). Disruption of Nf2- and Yap overactivation-results in abnormal glial structures and abnormally high levels of Slit2, with associated callosal agenesis as well as abnormal hippocampus commissure (Figure 2D). Yet, Nf2 disruption also affects the differentiation of glutamatergic guidepost neurons which may contribute to the observed callosal agenesis. The specific roles of midline glia vs. those of neuronal guideposts neurons in the formation of these axon commissures remain to be better distinguished. Otherwise, whether additional guidance cues to Slit2 are provided from the midline glial scaffolds, to guide callosal and hippocampal commissures remains to be investigated in more detail (Table 1). These midline glia guideposts also express the repulsive factor Draxin (Islam et al., 2009). This cue, initially identified from cDNA libraries of midline structures and commissural neurons of the chick, was shown to affect the pathfinding of commissural axons in the forebrain and the spinal cord, in the chick and the mouse. Recently, Draxin was also implicated in the proliferation and intercalation of the migrating *midline zipper* glia (Morcom et al., 2021a). In this context, Draxin is detected in glial cell progenitors and migrating glial cells and is thought to act autonomously. Yet, in this and other contexts, Draxin is detected both in midline glia and commissural neurons, and its cell-specific roles remain to be addressed in detail in different contexts.

In some contexts, guidance cues associated with glial scaffolds primarily drive the morphogenesis of the glial scaffolds themselves. For example, DCC and Netrin are expressed in the radial glia of the *midline zipper* and its progenitors and are required for appropriate morphogenesis of glia in the *midline zipper*. Loss of DCC function results in the absence of the glial processes in the ventricular zone, with subsequent effect in the formation of the corpus callosum (Morcom et al., 2021b). Such roles of axon guidance factors for glial cell morphogenesis are context-dependent. While the structure of the *glial wedge* and the morphology of its radial glial processes are disrupted upon loss of DCC receptor, DCC is not detected in radial glia of the neocortex (Morcom et al., 2021b). Thus, whether axon guidance cues acting in glia primarily drive axon pathfinding or morphogenesis of the glial scaffolds should be defined in every context. Besides, the architecture of the midline glia structures may also provide a physical barrier or scaffold for commissural axons, in addition to the expression of guidance cues for commissural navigation.

The anterior commissure is another major forebrain axon tract, which passes through the structure of the *glial tunnel*. This glia tunnel-like structure develops on both sides of the tract, while the anterior commissure axons begin midline crossing (Cummings et al., 1997; Pires-Neto et al., 1998). Recently, astrocyte glia were shown to populate this glial tunnel and the white matter of the anterior commissure and to impact its formation. These astroglia are regulated by Nkx2.1 and generated from three germinal regions of the ventral telencephalon, much earlier than generally accepted. Nkx2.1-derived astroglia and Nkx2.1-derived GABAergic interneurons act synergistically to regulate axon pathfinding of the anterior commissure, as demonstrated by selective cell ablation strategy (Figure 2D). These two Nkx2.1-derived cell populations mediate axon guidance of the anterior commissure through the expression of the repellent cue, Slit2 (Minocha et al., 2017). Glial cues affecting the anterior commissure remain to be further investigated.

The cortex is another major forebrain neuropil where glia have important functions in assembly. *Cortex radial glia* were suggested early to provide a scaffold for neuronal migration (Rakic, 1971). Yet their potential role in axon guidance was not functionally addressed until very recently. In parallel to the identification of midline glia structures, cortex radial glia were proposed early to assist callosal axons in finding their correct targets in the contralateral cortical plate, after midline crossing. These early light microscopy and ultrastructural studies in the sensorimotor cortex of neonatal hamsters highlighted that axons of callosal afferents are tightly associated with radial glia processes and their growth cones did not extend beyond associated radial glia fibers (Norris and Kalil, 1991). For long, the underlying molecular mechanisms of the radial-glia communication with axons remained unknown. Three decades later, radial glia (described also as BLBP-positive neural stem cells) in the ventricular zone of the medial ganglionic eminence were shown to guide corticospinal and other axons, at the junction between the striatum and globus pallidus. This radial glial functional role depends on the atypical RHO GTPase RND3 and its GTPase-activating protein partner that regulate actin cytoskeleton and glial fiber organization within radial glial neural stem cells (Kaur et al., 2020). Such function appears specific to the medial ganglionic eminence, and is not observed in radial glial fibers in the dorsal forebrain and the lateral ganglionic eminence. Recent work suggests that intermediate cortical progenitors, the lineage descendants of radial glial progenitors, may also be involved in axon guidance. They express guidance factors and receptors, including Netrin receptors, Plexins, Semaphorin, and Neuropilin (Bedogni and Hevner, 2021). The underlying molecular mechanisms of the communication between radial glia and axons in the cortex remain largely unknown to date.

### Guidance by glia in the mouse visual system

In the mammalian visual system, the organization of radial glia and their relationships with growing axons were first characterized in early light and electron microscopy studies (Guillery and Walsh, 1987; Hutchins and Casagrande, 1990). Axons of *retinal ganglion cells* (RGCs) exiting the retina, extend through the optic nerve to the optic chiasm, a complex intermediate choice point at the midline. There, some RGCs turn and grow into an ipsilateral optic tract while others cross the midline and enter the contralateral optic tract, to finally innervate the vision-processing targets in the thalamus (dorsal lateral geniculate nucleus) and superior colliculus (Figure 1E). This axon decision for midline crossing is regulated by cues presented by *optic chiasm radial glia* (Mason and Sretavan, 1997). Radial glia at the optic chiasm express Ephrin-B2 (Williams et al., 2003) and Nogo protein (Wang et al., 2008), to selectively repel axons of the ipsilateral optic tract that express EphB1 and Nogo receptors. Axons of the nasal and ventral temporal retina, present differential expression of EphB1 and Nogo receptors and navigate different paths (Herrera et al., 2004; Wang et al., 2017). The former, with lower receptor expression, cross the midline and the latter, with higher receptor expression, are repealed and turn to the ipsilateral optic tract (Figure 1E). Radial glia of the optic chiasm also express guidance cues Semaphorin 6D and Nr-CAM, which interact with Plexin-A1 and Nr-CAM on contralateral RGCs. This interaction inverts the repulsive action of Sema6D into a growth-promoting action of Sema6D when acting in combination with Nr-CAM and Plexin-A1. Through this complex molecular signaling, radial glia in the optic chiasm implement the contralateral projection of RGCs (Kuwajima et al., 2012). After crossing the optic chiasm, the crossed and non-crossed optic nerve tracts extend to the optic tectum (known as superior colliculus) or branch off to the lateral geniculate body of the thalamus to reach the occipital cortex. Radial glia in the optic tectum appear to express SKI and Furin convertases, that can generate RGMa fragments to bind Neogenin in the RGC axons and regulate proper axon targeting (Tassew et al., 2012). Disruption of any of these signals results in abnormal axon navigation in the optic chiasm (Figure 2E). Whether these guidance factors contribute functions from glial cells specifically for guidance or also can affect the architecture of radial glial cells prior to or in parallel to axon guidance remains unknown in these contexts and could be addressed with conditional, glial-specific manipulations and investigations.

### Guidance by glia in the mouse spinal cord

The commissural axons in the spinal cord have proved to be a key context for early functional studies of complex guidance decisions. Their navigation exemplifies well the complexity of integrating signals from numerous guidance cues (Colamarino and Tessier-Lavigne, 1995). Commissural axons extend for long distances away from their cell bodies, and encounter, sometimes simultaneously, numerous repulsive or attractive cues along their trajectory. They first appear to be repelled by the dorsal tissues of the neural tube, the *roof plate*. They then navigate in a circumferential ring along the pia surface, toward and across the midline, following cues expressed by intermediate targets along their way (Figure 1C). Before crossing their midline, the *floor plate*, axons are attracted by chemo-attractants including Netrin, Shh, and VEGF. When reaching the floor plate, axons stop responding to attractive cues and start responding to repulsive cues such as Sema3B and Slits to get expelled from the midline. These cues prevent axon recrossing and allow their growth to the contralateral side of the neural tube, where axons turn rostrally and extend toward the ventrolateral funiculus (Kolodkin and Tessier-Lavigne, 2011). Navigating commissural axons are affected by different glial cell sources along their way; roof plate cells that have suggested glial cell origin, the radial glial neural progenitors in the ventricular zone and the midline glia of the floor plate (Figure 1C).

The roof plate of the mouse neural tube was implicated rather early in axon repulsion. Commissural axons start growing ventrally, avoiding early the roof plate. Roof plate cells express bone morphogenetic proteins (BMPs) and GDF7 that can act as chemorepellents *in vitro* while *in vivo* they can orient the early trajectory of commissural axons toward the pia and ventral neural tube (Butler and Dodd, 2003). Specifically, BMP7 expressed by roof plate cells activates the cofilin regulator Lim domain kinase 1 (Limk1) *in vivo*, to control the rate of commissural axon extension and guide them ventrally (Phan et al., 2010). Roof plate cells also express the repellent Draxin which may also act on commissural neurons, although upon Draxin disruption commissural axons present defects in the level of the floor plate (Islam et al., 2009). Expression of these cues remains to be studied in single-cell resolution. Yet, radial glial cells may contribute to the expression of these roof-plate repellent cues, as they comprise at least part of the roof plate (Kridsada et al., 2018; Shinozuka and Takada, 2021). These *Nestin-positive stretched roof plate cells* also express Wnt signals which are important for the specification of the commissural neurons (Muroyama et al., 2002; Chizhikov and Millen, 2005).

The floor plate of the mammalian neural tube was implicated early in commissural axon pathfinding, first through the functional investigation of Netrin1 and of other attractive and repulsive cues later. Netrin-1 transcripts were highly detected in floor plate cells and the ventricular zone at the time of early axonogenesis, while the protein was suggested to act long-range function for chemotropic attraction in the spinal cord and other forebrain commissures (Kennedy et al., 1994; Serafini et al., 1996). The floor plate cells also express the secreted morphogen Sonic hedgehog (Shh), which provides chemoattraction to pre-crossing commissural neurons. Shh is also implicated in the fate specification of spinal cord neurons along the dorsoventral axis, but its role in axon guidance is thought to be independent of fate regulation (Charron et al., 2003). Both roles require Smo receptor, yet downstream components differ significantly (Yam and Charron, 2013). Netrin from floor plate cells is shown to cooperate with the secreted morphogen Sonic hedgehog (Shh) for the attraction of commissural axons (Wu et al., 2019). Floor plate cells also secrete the prototypic angiogenic factor VEGF to attract commissural axons, through its neuronal receptor Flk1(de Almodovar et al., 2011).

After the attraction of pre-crossing axons, the expression of several repulsive cues is attributed to floor plate cells, to guide post-crossing axons (Table 1). The Slits, were early-characterized chemo-repulsive cues, detected in a subset of midline glial cells of the developing CNS and deposited on the traversing axons (Rothberg et al., 1990). While the simultaneous loss of Slit1 and Slit2 proteins does not cause disruption of commissural axons, loss of all three Slit proteins results in extensive defects in the navigation of post-crossing commissural axons, with failure in midline crossing or defective recrossing (Long et al., 2004). Moreover, Slit1 and Slit2 redundantly affect the longitudinal tracts of the spinal cord, through the redundant role of Robo1 and Robo2 (Farmer et al., 2008). Dystroglycan is also found enriched in floor plate cells and regulates the localization of Slits in the floor plate and basement membrane (Wright et al., 2012). Absence of dystroglycan results in highly defective post-crossing of commissural axons toward the funiculus, with only minor disruptions in glial structures. In addition, Ephrin-B3 is expressed in floor plate cells and is implicated in commissural axon guidance at the ventral midline, through its neuronal Eph receptors (Kadison et al., 2006). Embryonic floor plate cells of the spinal cord also express repulsive Semaphorin3B, which can act through Neuropilin Nrp2 receptors on the post-crossing commissural neurons, to prevent them from recrossing the midline (Zou et al., 2000). Yet, intriguingly, Nrp2 is also reported to be expressed by floor plate cells and required for the pre-crossing pathfinding of commissural axons. So, while Neuropilin can act as a Semaphorin receptor in neurons, Nrp2 from the floor plate may act as a molecular sink to sequester repellent Semaphorin, preventing premature repulsion of pre-crossing axons (Hernandez-Enriquez et al., 2015). The floor plate is also reported to activate the responsiveness of axons to its repulsive Semaphorins through expression of additional factors. Intriguingly, the attractive cue Sonic Hedgehog was first proposed to activate the responsiveness of post-crossing axons to Semaphorin-mediated repulsion (Parra and Zou, 2010). Instead, more recent studies implicated the neurotrophic factor GDNF from the floor plate in axon responsiveness to Semaphorin repulsion, acting with the second floor-plate cue NrCAM (Charoy et al., 2012). Finally, floor plate glial cells also express Nogo-B to repel post-crossing axons. Axons arriving at the floor plate upregulate their Nogo receptor and are repelled out of the midline by Nogo-B, through contact-mediated and diffusible mechanisms (Wang et al., 2017).

Thus, several attractive and repulsive cues with key roles in the development of neural tube cell differentiation and navigation are provided by the floor plate, that contains glial cells (Figures 1C, 2C). However, the floor plate origin(s) remain controversial and its cell composition and fate is heterogeneous morphologically and molecularly, along both the anterior–posterior and the rostrocaudal vertebrate axis. Several studies find that “ependymal” (glial-like) cells are mixed with neuroblasts and differentiated neurons in the midbrain floor plate while an ependymal floor plate is less clear in the forebrain compared to the spinal cord. Several key questions remain open, including how many different populations of floor plate cells exist and are these functionally, as well as molecularly and ontologically, distinct (Placzek and Briscoe, 2005). Since the floor plate is not composed solely of glial cells, the glial cell contribution to the above functions contrary to possible neuronal roles remains to be clarified experimentally.

Another glial cell population that commissural axons meet in their path is the radial glial cells of the ventricular zone. Before any proof of their role in the formation of spinal cord axonal tracts, their contribution to axon navigation was suggested due to their organization during the peak periods of axonogenesis (Barry et al., 2013). Distribution of the radial glial cell scaffold coincides with the initial patterning of axon paths while the maturation of axon tracts coincides with the decline of the radial glial scaffold and their transformation into astrocytes. The radial glial cells scaffold may function to compartmentalize the white matter and allow patterning of the embryonic spinal cord. Recently, radial glia neural progenitors in the ventricular zone of the developing mouse neural tube were shown to drive the navigation of commissural axons along the pia surface toward the midline (Dominici et al., 2017; Varadarajan et al., 2017). These bipolar cells, extending from the ventricular surface to the pia, express and transport Netrin to their endfeet, which contact the laminin-positive pia surface (Figure 1C). The mechanism to transport Netrin in radial-glial endfeet remains to be identified. Overall, Netrin appears to have limited diffusion from the ventricular zone and is transported on the surface of commissural axons along the pia (Dominici et al., 2017; Varadarajan et al., 2017; Figure 2C). The radial glia of the ventricular zone may guide commissural axons through haptotaxis, a mechanism similar to the roles of membrane-tethered Netrin in *Drosophila* axon guidance (Brankatschk and Dickson, 2006; Salecker et al., 2012). Netrin1 in the mouse pia surface may orient the navigation and fasciculation of early “pioneering” commissural axons in the neural tube (Varadarajan and Butler, 2017). Here the notion of pioneers refers to early growing axons, while distinct molecular characteristics of these “pioneers” or their functional importance for “follower components” remain to be characterized. The suggested haptotactic function of Netrin from radial glia neural progenitors in the hindbrain and spinal cord seemed to upturn a decades-old idea about long-range guidance roles of Netrin from the floor plate. However, earlier studies already suggested a short-range activity of floor plate Netrin toward commissural axons (Matise et al., 1999). In spinal cords missing the floor plate specialization in *Gli2* mouse mutants, commissural axons reach the midline and the Netrin mRNA is still detected in a dorsally decreasing gradient. Thus, the floor plate appears dispensable for long-range cues to the commissural axons. Ventral midline cells are still recognized for their role in midline crossing and the rostral polarity guidance of commissural axons, although the glial-specific roles remain to be established (Wu et al., 2019). Certainly, radial glia of the ventricular zone drive commissural axon pathfinding.

### Guidance by glia in the mouse PNS

The effect of glia in the guidance has also been examined recently in peripheral neurons of the olfactory and the auditory system. In the developing olfactory system, sensory axons navigate from the olfactory placode to the forebrain, generating odortopic maps of mostly homotypic olfactory axons, expressing the same odorant receptor. Prior to this sorting, olfactory sensory neurons initiate navigation toward the olfactory bulb, following *olfactory ensheathing glial cells*. Developing olfactory ensheathing cells migrate ahead of the axons to establish a path and can secrete, among other, the Wnt inhibitor Frzb that affects basement membrane breakdown and axon targeting (Rich et al., 2018). The pre-target axon sorting, during the formation of the olfactory map, is also affected by the activity of the Neuropilin-1 guidance receptor and its repulsive ligand Semaphorin-3A. These are expressed in olfactory sensory neurons in a complementary manner and their loss results in perturbed axon sorting. Interestingly, Semaphorin3A is also expressed in ensheathing glia but its role remains to be examined. Recently, ensheathing glial cells were also implicated in the pathfinding of spiral ganglion neurons, in the mouse cochlea (Druckenbrod et al., 2020). Spiral ganglion neurons extend toward their targets, the hair cells, through an extremely heterogeneous environment of the inner ear. Spiral ganglion neurons with different positions seem to utilize different mechanisms for navigation, growth and fasciculation along glial or neuronal scaffolds. However, depletion of glial cells in the cochleae does not completely disrupt innervation, which seems to also depend on neuron–neuron fasciculation. Glial precursors may synergize with neurons to improve the efficiency of innervation in the cochlea. The molecular nature of these glia–neuron interactions and glia–neuron synergies remains to be identified.

## Axon guidance by astroglia: insights from the chick

### Guidance by glia in the chicken CNS

The chick was one of the early vertebrate models to study axon guidance, in parallel with the mouse. Early studies with *retinal ganglion cells* and *radial glial cells* of the chick retina demonstrated a differential outgrowth of axonal or dendritic processes on different radial glial cell compartments. Specifically, axons or dendrites were shown to grow preferentially in radial glial cell endfeet or cell somata, respectively (Bauch et al., 1998). Remarkably, these findings occurred 2–3 decades before any proven radial glia-mediated axon guidance in mouse circuits. Numerous mouse studies now contribute examples of suggested glial roles in axon pathfinding. Yet, from early on until today, the use of the chick contributed some unique details in the identification of floor plate guidance cues and the characterization of underlying molecular mechanisms *in vivo.* For example, studies in the chick developing spinal cord proposed the floor plate cue NrCAM as a ligand to neuronal Axonin, for proper growth cone guidance and midline crossing of commissural axons (Stoeckli and Landmesser, 1995). Floor plate activity of NrCAM was also recently identified in the mouse, as discussed above. Recently, chick studies revealed new roles of the Nectin-like molecule Necl3/SynCAM2, required in the floor plate for post-crossing commissural axon guidance (Niederkofler et al., 2010). Besides identifying new floor plate cues, recent studies in the chick also contribute to a mechanistic understanding of the primary role of glial cell architecture in defining guidance. Floor plate glia with progenitor-like, bipolar morphology and basal process endfeet on the basal lamina, present clusters of specific guidance cue fragments in their complex endfeet ramifications that mark the entire floor plate navigation path. The activity of proprotein convertase PC2 contributes to these ligand patterns. Growth cones establish contact with these glial cell surfaces during midline crossing. The clusters of Slit and Semaphorin3B fragments on these endfeet produce constraints protecting the growth cones from the risk of aberrant deviations due to active exploration of other cues in their environment (Ducuing et al., 2020).

The mechanistic underpinnings for the action of floor plate Semaphorin in commissural axon guidance were extensively studied, in parallel in the mouse and chick models. These studies revealed remarkable mechanistic details about dynamic receptor sorting on growth cones, and dynamic sensitization of commissural axons to midline cues. They revealed that floor plate Semaphorin signal is sensed differentially by pre- and post-crossing commissural axons due to the dynamic action of Neuropilin-2 and Plexin-A1 (Pignata et al., 2019). Moreover, a dynamic spatiotemporal sorting of Plexin and Robo receptors on commissural-axon growth cones allows the post-translational orchestration of a dynamic sensitization to midline Slit and Semaphorin cues, to allow dynamic changes in growth-cones behavior (Nawabi et al., 2010). Despite these findings being remarkable, here I focus on glial regulation and will not detail the dynamic sensing of neuronal receptors.

## Discussion

### Emerging themes of glial-mediated axon guidance across species

Surveying glial cell functions in axon navigation suggests that glial cells can employ any of the mechanisms of chemotaxis or haptotaxis for axon attraction or repulsion, depending on the context. This survey suggests that certain glial cell mechanisms of axon pathfinding span across model organisms. For example, some guidance cues are employed by distinct invertebrate and vertebrate glial cells, suggesting emerging themes shared across contexts (Table 1). Netrins function in most known contexts of glial-mediated axon guidance. They act for attraction in the mouse spinal cord -from ventricular zone radial glia and floor plate glia- and in the midline glial structures apposing forebrain axon commissures. They also act in the nematode brain neuropil and fly ventral cord. Similarly, Semaphorins act across invertebrate and vertebrate models, from the mouse floor plate, optic chiasm and olfactory ensheathing cells, to the fly and nematode’s brain glia. Certain Slits are also important for repulsion in the mouse floor plate, brain glial wedge & indusium griseum as well as the midline glia in the fly ventral cord. Other cues acting in more than one contexts, include the Shh and Frizzled, each shown to act in the vertebrate floor plate and zebrafish forebrain commissures. Otherwise, the transmembrane protein Flamingo acts in the fly ventral cord and the nematode brain glia. Interestingly roles for protein convertases and RhoGAP proteins have also been identified across models, from the mammalian to the nematode CNS.

Otherwise, disrupting the development of glial structures also affects the assembly of the associated axons, and certain underlying mechanisms are shared across models (Table 1). For example, the transcription factor Lhx2 and homologs of the NKX-family affect specification of glial cells that mediate axon guidance in several contexts. Nkx2/NKX-6 derived astroglial cells in the nematode brain guide pioneer and follower axons. Nkx2.1-derived astroglia in the mouse ventral telencephalon, guide the anterior commissure. Nkx2.2a-positive glial cells in the zebrafish ventral spinal cord guide axons in spinal cord exit points. The Nkx2.9 transcription factor in the mouse spinal cord, expressed in neural progenitors, is also required for motor axon exit but it is suggested to act cell-autonomously in neurons in that context (Bravo-Ambrosio et al., 2012). On the other hand, Lhx2 regulates specification of glial cells in the zebrafish spinal cord, affecting commissural axons while the mouse Lhx2 regulates development of the glial wedge and indusium griseum and subsequently affects the formation of the corpus callosum. Besides, secreted Slits cues also regulate development of the glial cells driving axon guidance in several contexts. For example, certain Slits are implicated in the development and migration of glial cells in the fly midline as well as in the zebrafish CNS forebrain commissures. Overall certain glial cell contributions in axon navigation and their underlying mechanisms may be conserved across species.

### Glial cell roles in the pathfinding of pioneer axons

The blueprint hypothesis implies that glial scaffolds not only guide certain axonal components but rather initiate circuit assembly by guiding pioneer axons, yet very few case studies demonstrate this. Glial cells in *Drosophila* appear rather dispensable for pioneer axon paths of the posterior commissure, ventral cord commissural and longitudinal tracts, and other embryonic primary axon tracts (Klämbt et al., 1991; Hidalgo et al., 1995). In zebrafish, pioneer axon growth cones of PNS sensory neurons initially extend together with glial cells but are seen to navigate without glial cell contact when crossing the PNS/CNS interface to enter the CNS (Nichols and Smith, 2019). Regulation of pioneer axons by glial cells was first proposed in the grasshopper limb bud, where *segment boundary glial cells* appear functionally indispensable for navigation of pioneer axons (Bastiani and Goodman, 1986). Roles of astroglial cells in axon navigation of defined functional pioneer axons are identified by *in vivo* functional assays in the *C. elegans* embryonic brain-like neuropil. There, brain-neuropil-associated CEPsh glia are required for the correct pathfinding of both pioneer and follower neurons as identified by cell ablation and genetic studies (Rapti et al., 2017). Specifically, these astroglial cells utilize Netrin and other unknown cues to drive the correct pathfinding of pioneer axons and distinct cues to guide follower axons. Otherwise, radial glial cells of the mouse ventricular zone are suggested to guide the “first” mouse commissural axons but the identity of these first axons and their functional significance for the commissural bundle remain to be functionally defined (Varadarajan and Butler, 2017). Overall, identifying interactions of astroglial cells with pioneer neurons and glial mechanisms of pioneer axon pathfinding ought to be an important next research aim of this field. This would also allow to shed light into if and how glial cells can themselves pioneer the architecture of early developing neuropils. These studies should benefit from single-cell-resolution studies and cross-species investigations between genetically tractable model organisms since molecular identities of pioneer neuron are often unknown in complex neuropils but identified in neuropils with less components.

### Glial cell development and axon guidance: chicken-and-the-egg problems

It is often not clear which regulation comes first: a regulation of glial cell development or migration affected by neurons or a glial-mediated regulation of axon pathfinding, or even other glial cell functions in neuronal physiology, neurogenesis, axon ensheathment or metabolic support. In several cases, the development of glia and neurons appears co-dependent. For example, embryonic axons of the fly sensory (Futsch-positive) neurons have abnormal axons or may die in the absence of associated glia, while they are themselves required for glial cell formation and migration (Sepp and Auld, 2003). Time-lapse and cell ablation studies in the fly pupal wing suggest that neuron–glia interactions can influence the migratory directions of glial cells (Aigouy et al., 2004). In the developing visual system of flies, glial cells provide positional information for photoreceptor axon guidance, as discussed above, yet, glial cell migration itself depends on these axons. Photoreceptor axons originating in the vicinity of glial progenitors, provide a scaffold for targeted migration of glial cells in specific highways in the optic lobe (Dearborn et al., 2002). Importantly, glial molecular cues with roles in axon pathfinding often have roles in regulating the development of glial cells themselves. Fly proteins Netrin and Slit are initially important for the development and migration of glia or glial cell precursors, with concomitant implications for the pathfinding of midline commissural axons (Rothberg et al., 1990; Von Hilchen et al., 2010). Sometimes, the effect of glia on axons may be an indirect consequence of glial roles in neuronal birth and survival. The *Drosophila* chloride channel ClC-a is expressed in cortex glia in the stem cell niche, mediates ionic homeostasis, and regulates neurogenesis and gliogenesis, also generating glial cells that guide photoreceptor axons (Plazaola-Sasieta et al., 2019). Glial ensheathment of axons may also influence axon pathfinding in indirect ways. For instance, after initial commissure formation, midline glia wrap commissural axons in part through the adhesion of glial protein Wrapper and neuronal Neurexin IV. In the absence of this axonal wrapping, glia migrations and consequently the axon fasciculation patterns are affected (Noordermeer et al., 1998; Stork et al., 2009; Wheeler et al., 2009). Thus, it is important to distinguish primary, direct roles and mechanisms of glial cells in axon pathfinding or other cellular events required for neural circuit development and architecture. Such clarity is feasible in *C. elegans*, where astroglial cells appear largely decouple from neuronal birth and viability, possibly due to the deterministic lineage of the animal (Sulston et al., 1983).

Factors acting from glial cells for axon pathfinding are also recognized for contributions to astroglial cell development in some cases, as discussed above. Netrin and Slit cues regulate glial cell development and migration in the fly (Rothberg et al., 1990; Von Hilchen et al., 2010). Slit2-Slit3 and Frizzled-3a control patterning of zebrafish midline glia, while transcription factor Lhx2 affects the positioning of GFAP-positive glia in zebrafish and formation of the mouse glia in the wedge and indusium griseum (Lyall et al., 2015). Formation of glial cells in the glial wedge and indusium griseum, mouse glial structures opposed to brain commissures, are also affected by Nf2-Yap and Fgfr1 signaling (Ohkubo et al., 2006; Smith et al., 2009). Mouse transcription factors Nkx2.2 and Nkx2.9 regulate the development of floor plate glial cells thus affecting commissural axon guidance (Holz et al., 2010). Disruption of the mouse Netrin receptor DCC affects the defects in astroglial morphology and migration (Morcom et al., 2021b). Yet, in contrast to regulators of fate specification and migration of glial cells, mechanisms driving their morphogenesis remain even more understudied. And yet this architecture may be key for axon pathfinding, either through physical interactions of adhesion or by presenting specific subcellular localization of membrane-bound guidance factors, such as the aforementioned Slit and Semaphorin3B clusters on endfeet of radial glial cells in the developing mouse spinal cord (Ducuing et al., 2020). Thus, studying how astroglial cells establish their morphological cell architecture should be another important forefront in the investigations of neuropil development and axon navigation.

Several approaches and technical advancements can accelerate research to provide answers in open questions highlighted throughout the different systems of study. Identifying molecular factors that underly glial roles in axon pathfinding can benefit from recent transcriptomic studies that can inform on the (glial-) cell specific transcriptional landscape. Combining this knowledge with genetic and cellular investigations *in vivo*, can identify new glial cell molecules with unknown functions in axon guidance in different neuropils. Cell perturbations or gene manipulations in single-cell or single-cell-type resolution, such as conditional knock-out animals, are key to distinguish glial-specific roles. Moreover, systematic real-time imaging *in vivo* or *ex vivo* of both neurons and glial cells in each studied context is important to describe the primary and secondary defects of cellular and genetic perturbations. Recent technological advances in animal models for investigation of glia–neuron crosstalk enable manipulating fluorescently-labeled cells or tracing their cellular processes *in vivo.* Combined efforts of cell-specific knock-out/expression studies and advanced imaging approaches should enable progress in these directions. Last but not least, cross-species investigations will facilitate addressing open questions; leveraging the knowledge achieved in genetically-tractable model systems, with less components and single-cell resolution studies, to perform comparative studies in more complex circuits, is key.

### Glia-mediated axon pathfinding underlying diseases

Abnormalities in the carefully orchestrated steps required to guide axon navigation and neural network architecture are thought to result in neurodevelopmental disorders. Abnormal development of specific axonal commissures or astroglial cell architecture, and mutated cues of axon guidance sometimes underly these pathologies. Dysfunction of guidance genes of the Netrin, Semaphorin, Ephrin pathways are associated to various neurodevelopmental and neuropsychiatric disorders. For example, 3%–5% of patients examined for neurodevelopmental disorders suffer dysgenesis or agenesis of the corpus callosum (Jeret et al., 1985; Bodensteiner, 1994). Corpus callosum dysgenesis is associated with pathogenic variants of human DCC, the receptor of glial Netrin driving key axon pathfinding decisions in the brain. Both family members and unrelated individuals carrying DCC pathogenic variants have high frequency of associated callosal commissure malformations (Jamuar et al., 2017; Marsh et al., 2018) and loss-of-function mutations in *DCC* underline the “split-brain” syndrome with disorganized axon tracts or loss of callosal commissure (Jamuar et al., 2017). Heterozygotes pathogenic variants of Netrin1 or its receptor DCC also underly the pathology of congenital mirror movements (CMM) that presents involuntary movements on one side of the body, that mirror voluntary movements on the opposite side (Srour et al., 2010). Genetic variations or disruption of Netrins (*NTNG1*/*2*) are also linked to bipolar disorder, schizophrenia and Rett syndrome (Woo et al., 2009). Besides, the second Netrin receptor UNC5 and its abnormal cleavage is associated to neurodegenerative diseases, including late-onset Alzheimer’s disease (AD) and Parkinson’s disease (PD; Wetzel-Smith et al., 2014; Chen et al., 2021). The Slit-Robo pathway is also associated with neuropshychiatric diseases. Mutations in the ROBO3 receptor are associated with axon midline crossing defects in the hindbrain, in patients with horizontal gaze palsy with progressive scoliosis (HGPPS) while *de novo* mutations in SLIT2/3 are identified in schizophrenia patients (Jen et al., 2004; Gulsuner et al., 2013). Sex differences in protein level regulation of SLIT is linked to abnormal dendritic arborization and sex-specific susceptibility to depression, through unclear mechanisms (van der Zee et al., 2022). Several neurodevelopmental and neurodegenerative diseases are associated with the Semaphorin and Ephrin pathways. Heteroinsufficiency of SEMA3A or its receptor PLXNA1 is implicated in Kallmann syndrome, a genetic disorder with neurological defects in odor detection (Van Battum et al., 2015). EPHA1 is a risk gene for late-onset AD while *EPHA4* is proposed to be a disease modifier gene for amyotrophic lateral sclerosis (ALS; Van Hoecke et al., 2012; Karch and Goate, 2015). Pathogenic variants of DCC, Robo, and other guidance cue receptors are also associated with autism spectrum disorders [ASDs; as reviewed in Bedogni and Hevner (2021)]. The gene locus of the cell adhesion molecule NrCAM, which regulates fasciculation of mouse brain commissures, shows distinct polymorphisms associated with ASDs (Pinto et al., 2010; Voineagu et al., 2011; Sakurai, 2012), while its knockout in male mice exhibits autism-related behaviors (Moy et al., 2009). Furthermore, axon guidance defects may bear links to epilepsy. For example, brain samples of epileptic patients show changes in the expression levels and spatiotemporal distribution of the guidance cue Slit2 in both neurons and astrocytes in temporal lobe epileptic foci (Fang et al., 2010). Many more guidance factors are associated with neurodevelopmental disorders, yet through unknown mechanisms [as reviewed in Zuchero and Barres (2015)]. Such studies provide a strong rationale to investigate more closely unknown defects of axon pathfinding in human neurodevelopmental disorders, by combining genetics and brain imaging.

Astroglia comprise more than half of the human brain cells and regulate various axon targeting decisions. The involvement of axon pathfinding defects in neurological disorders raises the question of which conditions may share an underlying etiology of abnormal astroglial cell development or function. Evidence has recently emerged for the involvement of astroglial development in neurodevelopment disorders, including autism spectrum disorders (ASDs). Studies of human ASD patient samples demonstrate elevated expression of the astroglial marker GFAP in the superior frontal, parietal, and cerebellar cortices (Laurence and Fatemi, 2005), as well as abnormal expression patterns of astroglial markers AQP4 and CX43 (Fatemi et al., 2008). More recent reports indicate that autistic patients present astrocytes with reduced branching length, processes number, and cell body sizes (Vakilzadeh and Martinez-Cerdeño, 2023). Otherwise, human pathogenic variants of DCC, associated with callosal dysgenesis, are unable to regulate cell morphology in human cell culture, and have a detrimental effect in astroglial cell shape and motility in mammalian model organisms *in vivo* (Morcom et al., 2021b). Overall, astroglia are also associated to epilepsies and other diseases through their function in synaptic regulation or their reactive physiology (gliosis/astrocytosis) that accompanies brain trauma. These contributions are reviewed elsewhere, since this survey focusing on the effects of astroglial-mediated axon guidance (Sase et al., 2018; Kim et al., 2020; Nagai et al., 2021). These findings provide the foundation for a closer examination of the link between astroglial development and architecture, and neurodevelopmental disorders including ASDs, schizophrenia, epilepsies or neurodegenerative disorders of the nervous system architecture.

Future strategies for modulating levels of guidance cues may be useful for restoring neural circuits. Modulating Netrin signaling may be valuable for alleviating pathologies of Alzheimer’s, Parkinson’s disease or neurodevelopmental disorders including bipolar disorder, schizophrenia and Rett syndrome. Specific guidance signaling pathways (such as the Slit/Robo pathway) can be neuroprotective while other morphogens (like WNTs) can create non-permissive environments. Manipulating the SLIT-ROBO signaling at neuron–glia interfaces may favor neural repair (Kaneko et al., 2018). Pharmacologically manipulating the Semaphorin signaling may enable functional recovery from brain damage during epilepsy (Abe et al., 2018). Overall, controlling neuron–glia communications to target neuronal guidance and rewiring may provide novel therapeutic strategies for neurodevelopmental/neuropsychiatric diseases and neural injuries. A precise and comprehensive understanding of glial roles of guidance pathways and neuron–glia crosstalk is key for such strategies.

### Non-glial cells driving axon pathfinding

Besides neurons and glial cells, the borders of axonal paths in the nervous system are also populated by other cells including ependymal and epithelial cells, pericytes, polydendrocytes, neural crest cells, mesodermally-derived microglia, and endothelial cells. Whether these cells act for the assembly of axon scaffolds and connections remains understudied. Early studies suggested that pigmented epithelial cells are involved in the development of the optic nerve, with melanin-producing stalk cells inhibiting the lateral spread of axonal growth within their territory, to control the patterning of optic fibers (Silver and Sapiro, 1981). More recently, more of these cell types were implicated in axon development in certain contexts. In the zebrafish embryo, neural crest cells in cooperation with neural tube border cells affect the afferent entrance of peripheral sensory neurons to establish topographical representation of sensory projections at the hindbrain level (Zecca et al., 2015). Sensory neuron positioning in the mouse head is also affected by the positioning of cranial neural crest cells, that migrate following Neuropilin 1 and 2 signaling (Schwarz et al., 2008). In the chick, Sema6A from boundary cap cells is required for the appropriate entry of sensory afferents into the dorsal spinal cord (Domanitskaya et al., 2010). In the mouse, multipotential polydendrocytes NG2 provide a favorable substrate for growing axons (Yang et al., 2006). Mouse vascular endothelial cells provide Semaphorin3 to inhibit axon growth of sympathetic neurons (Damon, 2006) while endothelial Neuropilin affects RGC axon organization (Erskine et al., 2017). Besides, the endothelial nerve growth factor in the optic chiasm is required for commissural axon chemoattraction (de Almodovar et al., 2011; Partyka et al., 2019) while neurovascular interactions direct axon growth in the injured mouse spinal cord (Partyka et al., 2019). In *Drosophila* and *C. elegans*, some epithelial and mesodermally-derived cells are also neighboring pathways of axons and glial cells in the nervous system. In *C. elegans,* epithelial cells are involved in the development of the ventral cord and navigation of its axonal pathways, as well as in the maintenance of glial cell architecture (Singhvi et al., 2023). Thus, non-neuron/glial cells may contribute important cues for axon navigation *in vivo*. Their cell-specific actions and underlying molecular mechanisms that may direct axon pathfinding await detailed investigation, especially in vertebrates.

## Author contributions

The author confirms being the sole contributor of this work and has approved it for publication.
